# Validation of the high-performance of pyrosequencing for clinical *MGMT* testing on a cohort of glioblastoma patients from a prospective dedicated multicentric trial

**DOI:** 10.18632/oncotarget.11322

**Published:** 2016-08-17

**Authors:** Véronique Quillien, Audrey Lavenu, François Ducray, Marie-Odile Joly, Olivier Chinot, Frédéric Fina, Marc Sanson, Catherine Carpentier, Lucie Karayan-Tapon, Pierre Rivet, Natacha Entz-Werle, Michèle Legrain, Emmanuèle Lechapt Zalcman, Guenaelle Levallet, Fabienne Escande, Carole Ramirez, Dan Chiforeanu, Elodie Vauleon, Dominique Figarella-Branger

**Affiliations:** ^1^ Centre Eugène Marquis, F-35042 Rennes, France; ^2^ Université Rennes 1, Faculté de Médecine, F-35043 Rennes, France; ^3^ INSERM CIC 0203, Université de Rennes 1, F-35043 Rennes, France; ^4^ Hospices Civils de Lyon, F- 69394, Lyon, Cedex, France; ^5^ Université de Lyon1, F-69622 Villeurbanne, France; ^6^ CHU Timone, F-13385 Marseille, France; ^7^ Faculté de Médecine Secteur Nord, F-13916 Marseille, France; ^8^ Sorbonne Universités UPMC Université Paris 06, INSERM CNRS, U1127, UMR 7225, ICM, F-75013 Paris, France; ^9^ INSERM U1084, Université de Poitiers, F-86021 Poitiers, France; ^10^ CHU de Poitiers, F-86021 Poitiers, France; ^11^ CHRU Hautepierre, F67098 Strasbourg, France; ^12^ EA 3430, Progression Tumorale et Microenvironnement, Approches Translationnelles et Épidémiologie, Université de Strasbourg, F-67000 Strasbourg, France; ^13^ CHU Caen, Département de Pathologie, F-14000 Caen, France; ^14^ CHRU de Lille, F-59037 Lille, France; ^15^ CHU de Rennes, F-35000 Rennes, France; ^16^ INSERM U911 CRO2, Université de la Méditerranée, F-13385 Marseille, France

**Keywords:** glioblastoma, prospective trial, MGMT, promoter methylation, pyrosequencing

## Abstract

**Background:**

The goal of this prospective multicentric trial was to validate a technique that allowed for *MGMT* promoter methylation analysis in routine clinical practice.

**Methods:**

The MGMT status of 139 glioblastoma patients, whom had received standard first line treatment, was determined using pyrosequencing (PSQ) and a semi-quantitative Methylation-specific PCR (sqMS-PCR) method, using both frozen and formalin-fixed paraffin-embedded FFPE samples. Eight participating centers locally performed the analysis, including external quality controls.

**Results:**

There was a strong correlation between results from FFPE and frozen samples. With cut-offs of 12% and 13%, 98% and 91% of samples were identically classified with PSQ and sqMS-PCR respectively. In 12% of cases frozen samples were excluded because they had a low percentage of tumor cells. In 5-6% of cases the analysis was not feasible on FFPE samples. The optimized risk cut-offs were higher in both techniques when using FFPE samples, in comparison to frozen samples. For sqMS-PCR, we validated a cut-off between 13-15% to dichotomize patients. For PSQ, patients with a low level of methylation (<= 8%) had a median progression-free survival under 9 months, as compared with more than 15.5 months for those with a level above 12%. For intermediate values (9-12%), more discordant results between FFPE and frozen samples were observed and there was not a clear benefit of temozolomide treatment, which indicated a “grey zone”.

**Conclusions:**

MGMT status can reliably be investigated in local laboratories. PSQ is the ideal choice as proven by strong interlaboratory reproducibility, along with threshold agreements across independent studies.

## INTRODUCTION

Since the introduction of temozolomide (TMZ) chemotherapy in the standard care protocol for glioblastoma (GBM) patients, *MGMT* promoter methylation analysis has become a crucial biological marker. As *MGMT* promoter methylation is recognized as a very powerful predictor of response to TMZ for newly diagnosed GBM patients, it is used to stratify or select patients in clinical trials [1, 2]. Moreover, the most recent recommendation in the EANO guideline was that *MGMT* testing should be standard practice specifically for elderly patients as, in combination with performance status, it could help clinicians selecting the appropriate treatment for these patients [3]. The routine implementation of *MGMT* testing to aid decision making in GBM patients raises complex issues, including the choice of the optimal molecular test (for review 1) [1].

The two most popular and validated techniques to assess MGMT status are, quantitative Methylation-Specific PCR (Q-MSP) [4–6] and pyrosequencing (PSQ) [6–12]. The Q-MSP technique that has been used in several international clinical trials determines the number of copies of methylated *MGMT*, which is then normalized to the number of copies of the *ACTB* gene. PSQ is a technique based on the principle of sequencing-by-synthesis that quantifies DNA methylation levels at individual selected CpG sites. Approximately, 30% of GBM patients are classified as “methylated” with Q-MSP [4, 5] as compared to about 45% with PSQ [6, 7, 10].

A crucial factor for a clinical setting method is a high degree of repeatability and reproducibility. To be reliable for a clinical use, a technique must display a high repeatability and reproducibility. Repeatability represents the degree of agreement among repeated measurements obtained for one identical sample, in one laboratory, on a single apparatus, with the same operator; while reproducibility signifies the degree of agreement among repeated measurements, obtained under different conditions, in different laboratories for one identical sample. Reproducibility indicates the robustness of the test, which is extremely important for techniques implemented in multiple laboratories. Additionally, it is important to consider the validation cut-off points. There is a span of *MGMT* methylation measurements in clinical samples, ranging from very low (limit of detection of the technique) to very high. To classify samples as “methylated” or “unmethylated”, occasionally a mathematical cut-off will be utilized. Authors have reported a bimodal distribution for *MGMT* methylation measurements with Q-MSP and considered a cut-off the minimum value between the two distributions [13]. Alternatively, other authors have based the cut-off on values obtained in non-neoplastic brain samples (mean of normal brain samples +/− two standard deviations) [8]. Intuitively, the optimal cut-off would be the minimal *MGMT* methylation that is able to suppress MGMT expression. This is able to be investigated by utilizing cell lines and comparing *MGMT* methylation and MGMT expression. However this biological cut-off would not take into account the complexity of GBM samples that may contain a variable number of non-neoplastic cells whose “unmethylated” *MGMT* DNA is extracted with that of tumor cells. This variable could lead to an underestimation of the level of *MGMT* methylation of the tumor cells [14]. This can be overcome by macrodissection of samples to ensure a high percentage of tumor cells. However, selecting an area rich in cancer cells can be challenging: recent studies have shown the difficulty to accurately assess the percentage of tumor cells [15, 16]. Moreover, in GBM samples non-tumor and tumor cells are often intermingled together. Thus, a compromise would be to establish on outcome-based cut-off that needs to be validated in multiple cohorts of patients, ideally prospectively.

In a previous study we demonstrated that PSQ was the most optimal technique among the five that were tested [10]. We have extended our analysis into a prospective study. 163 GBM patients were enrolled in 8 centers and MGMT testing was performed in each center on both frozen and FFPE samples. As PSQ requires a specific piece of equipment, which is not always available in molecular laboratories, we also investigated using an alternative fluorescent semi-quantitative methylation-specific PCR (sqMS-PCR) [17]. The goal of this study was to validate a method that allows for the quantification of *MGMT* methylation in routine clinical practice and to establish clinical cut-offs. We show that MGMT status can reliably be investigated in local laboratories. PSQ is the ideal choice as proven by strong interlaboratory reproducibility. We recommend a classification of MGMT promoter methylation status in three subgroups: “unmethylated” (0-8%), “methylated” (13-100%) and a grey zone for patients with intermediate values (9-12%).

## RESULTS

### Study population

**Figure 1:**
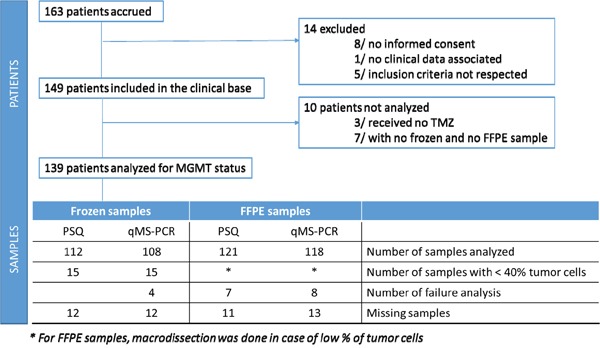
Prospective Multicentre Study Patient Profile

Among the 163 patients enrolled in the study, 14 were excluded due to non-compliance of the inclusion criteria. 10 additional patients were excluded because they had not received TMZ (n=3) or there was no sample to be tested (n=7) (Figure 1). Clinical patient characteristics are summarized in Table 1. A total of 119 (85.6%) patients died once the database was closed. The median PFS was 9.5 months (8.9 – 11.2; 95% CI) and the median OS was 20.0 months (18.3 – 22.6; 95% CI).

**Table 1:** Patient characteristics

Median age at surgery in years (range)	55.9 (23.0 – 71.0)
Gender, n	
Females	41
Males	98
Type of surgery, n	
Total resection	78
Partial resection	41
Biopsy	20
KPS, n	
90-100	41
70 – 80	76
< 70	20
Missing	2
Cycles of TMZ in adjuvant, n	
Median (range)	6 (0-30)

### Analysis of reproducibility

**Figure 2:**
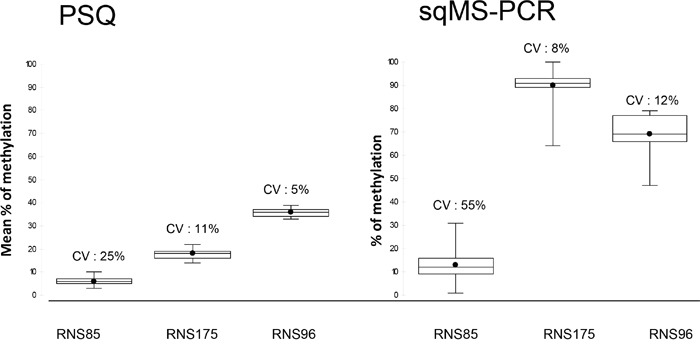
Box plot representation of results of the 3 controls (RNS85, RNS175 and RNS96) tested by pyrosequencing (PSQ) and semi-quantitative Methylation-specific PCR sqMS-PCR Each control was repeated at least in duplicate at each of the participating laboratories. The box plots depict the minimum and maximum values observed, the upper (Q3) and lower (Q1) quartiles (the length of the box represents the interquartile range), the median (identified by a line inside the box) and the mean (identified by the black point). Above each box the coefficient of variation (CV) estimates the interlaboratory reproducibility of the technique at the observed value.

As repeatability has been previously reported for the two techniques [10, 17], only reproducibility was tested in this study. DNA from 3 cell lines (RNS85, RNS96 and RNS175), were used as external quality controls, to assess interlaboratory reproducibility. Each control was tested in at least duplicate in the different participating laboratories. The mean values for RNS85 were 6% (range: 3-10%, n=22) by PSQ and 13% (range: 1-31%, n=20) by sqMS-PCR. The mean values for RNS175 were 18% (range: 14-22%, n=22) by PSQ and 90% (range: 64-100%, n=19) by sqMS-PCR. The mean values for RNS96 were 36% (range: 33-39%, n=22) by PSQ and 69% (range: 47-79%, n=19) by sqMS-PCR. RNS85 level of methylation was close to the limits of quantification previously published for PSQ (4%) [10] and sqMS-PCR (15%) [17]. The reproducibility CV was 25% (with PSQ) and 55% (with sqMS-PCR) for RNS85. The CV of RNS175 was 11% (PSQ) and 8% sqMS-PCR and 5% (PSQ) and 12% (sqMS-PCR) for RNS96 (Figure 2).

### Comparison of MGMT methylation results on FFPE and fresh frozen samples

**Figure 3:**
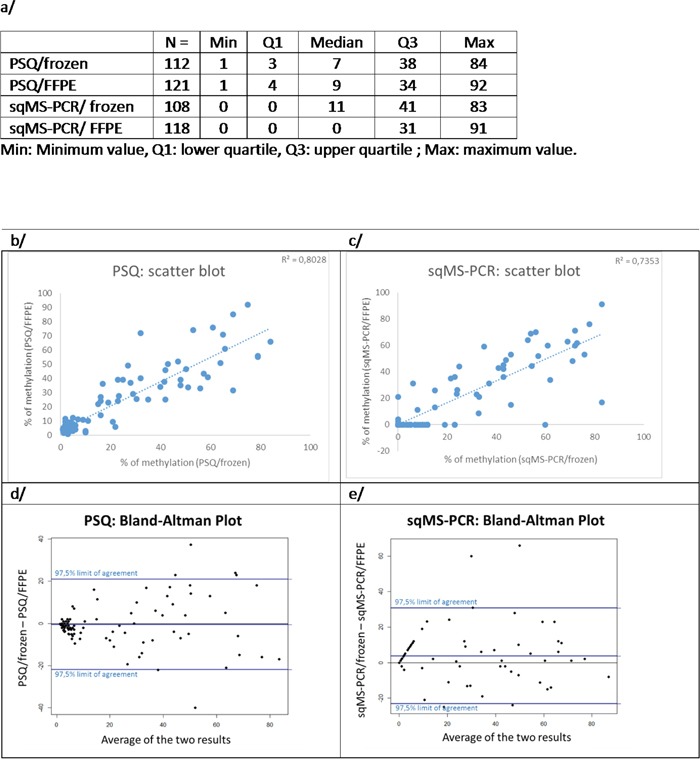
Comparison of results obtained on FFPE and frozen samples **a.** distribution of data according to the technique and the type of sample. **b-e.** agreement between frozen and FFPE samples for PSQ (b and d) and sqMS-PCR (c and e) analysis using Bland-Altman (d and e) or scatter plots (b and c).

Figure 1 shows that 12% (15/127) of frozen samples were determined to have less than 40% of tumor cells and therefore, were removed from this study. The PSQ method was shown to be feasible on all frozen samples available, in comparison to 4 failure analyses occurring when using the sqMS-PCR method. Failure analysis was more frequent in FFPE samples (7 and 8 cases respectively for PSQ and sqMS-PCR). The median percentages of methylation were 7% when using PSQ on 112 frozen samples (range 1-84%) and 9% on 121 FFPE samples (range 1-92%). Furthermore, the median percentages of methylation were 11% when using sqMS-PCR on 108 frozen samples (range 0-83%) and 0% for sqMS-PCR on 118 FFPE samples (range 0-91%) (Figure 3a). A strong correlation was observed for the 95 samples analyzed by the PSQ method, as well as, the 93 samples analyzed using the sqMS-PCR method with DNA extracted from FFPE and frozen samples (Figure 3b/3c).

### Predictive impact of different cut-off values of MGMT Methylation

Threshold values to separate low- and high-risk patients according to their outcomes were determined for each technique and type of sample. Table 2a displays the best cut-offs for OS as 6% for PSQ on frozen samples, 16% for PSQ on FFPE samples, 13% for sqMS-PCR on frozen samples and 23% for sqMS-PCR on FFPE samples. Additionally, Table 2b displays the best cut-offs for PFS. Optimized cut-offs were higher in both techniques when using FFPE samples compared to frozen samples, despite the lack of a systematic higher value being observed with this type of sample (Bland-Altman plot- (Figure 3)).

**Table 2:** Comparison of various prognostic impacts when evaluating MGMT promoter methylation using pyrosequensing (PSQ) and a semi-quantitative methylation-specific PCR (sqMS-PCRS) from frozen or FFPE samples

a/OS							
Type of analysis	type of cut-off	cut-off value	% of patients with a methylated MGMT promoter	HR	p	AUCROC	CHarrell
PSQ/FFPE	optimized cut-off (current series/FFPE samples)	16	39	0,22	<1,00E-06	0,70	0,71
PSQ/frozen	optimized cut-off (current series/frozen samples)	6	51	0,25	<1,00E-06	0,69	0,70
PSQ/frozen	optimized cut-off (previous series/frozen samples)	8	49	0,28	<1,00E-06	0,69	0,69
PSQ/FFPE	best level of concordance between frozen and FFPE samples	13	40	0,23	<1,00E-06	0,69	0,70
PSQ/frozen	best level of concordance between frozen and FFPE samples	12 or 13	44	0,29	<1,00E-06	0,69	0,70
PSQ/FFPE	best level of concordance between frozen and FFPE samples	12	41	0,24	<1,00E-06	0,69	0,70
PSQ/FFPE	optimized cut-off (previous series/frozen samples)	8	51	0,25	<1,00E-06	0,68	0,70
PSQ/frozen	optimized cut-off (current series/FFPE samples)	16	40	0,32	3,00E-06	0,68	0,69
qMS-PCR/FFPE	optimized cut-off (current series/FFPE samples)	23	31	0,24	<1,00E-06	0,68	0,69
qMS-PCR/frozen	optimized cut-off (current series/frozen samples) and best level of concordance between frozen and FFPE samples	13	45	0,35	1,20E-05	0,67	0,67
qMS-PCR/FFPE	best level of concordance between frozen and FFPE samples	12	40	0,3	<1,00E-06	0,67	0,67
PSQ/FFPE	optimized cut-off (current series/frozen samples)	6	59	0,31	<1,00E-06	0,66	0,68
qMS-PCR/FFPE	best level of concordance between frozen and FFPE samples	13	37	0,3	<1,00E-06	0,66	0,67
qMS-PCR/frozen	optimized cut-off (current series/FFPE samples)	23	38	0,46	8,87E-04	0,65	0,64

b/PFS							
Type of analysis	type of cut-off	cut-off value	% of patients with a methylated MGMT promoter	HR	p	AUCROC	CHarrell
PSQ/frozen	optimized cut-off for PFS (current series/frozen samples)	15	43	0,3	<1,00E-06	0,66	0,64
PSQ/frozen	optimized cut-off for OS (current series/frozen samples)	6	51	0,27	<1,00E-06	0,66	0,63
PSQ/frozen	best level of concordance between frozen and FFPE samples	12 or 13	44	0,31	1,00E-06	0,66	0,63
PSQ/frozen	optimized cut-off for OS (previous series/frozen samples)	8	49	0,32	1,00E-06	0,66	0,63
PSQ/FFPE	optimized cut-off for PFS (current series/FFPE samples)	19	38	0,3	<1,00E-06	0,66	0,63
PSQ/FFPE	optimized cut-off for OS (current series/FFPE samples)	16	39	0,31	<1,00E-06	0,65	0,62
PSQ/FFPE	best level of concordance between frozen and FFPE samples	13	40	0,31	<1,00E-06	0,65	0,62
PSQ/FFPE	best level of concordance between frozen and FFPE samples	12	41	0,32	1,00E-06	0,65	0,62
PSQ/frozen	optimized cut-off for OS (current series/FFPE samples)	16	40	0,37	1,40E-05	0,65	0,62
PSQ/FFPE	optimized cut-off for OS (previous series/frozen samples)	8	51	0,36	3,00E-06	0,64	0,61
qMS-PCR/FFPE	optimized cut-off for PFS (current series/FFPE samples)	23	31	0,31	1,00E-06	0,64	0,62
qMS-PCR/frozen	optimized cut-off for PFS (current series/frozen samples)	13	45	0,42	2,28E-04	0,64	0,60
PSQ/FFPE	optimized cut-off for OS (current series/frozen samples)	6	59	0,39	1,30E-05	0,63	0,61
qMS-PCR/FFPE	best level of concordance between frozen and FFPE samples	13	37	0,37	6,00E-06	0,63	0,61
qMS-PCR/FFPE	best level of concordance between frozen and FFPE samples	12	40	0,4	2,20E-05	0,62	0,60

In regards to each specific technique, different cut-offs were evaluated to determine the associated Hazard ratio (HR) and the level of significance (represented by the p value, which is to compare to 1.3/1000 with the multiple comparison correction of Bonferroni), after adjustment on age and Karnofsky score. The prediction errors were globally evaluated and reported as the Area Under the ROC Curve (AUCROC) and the Harrell's C index. Results are classified according to the AUCROC result, from the highest to the lowest value. The same analysis was done for overall survival (OS/a) and progression free survival (PFS/b).

To determine a cut-off that would be appropriate for both sample types, we tested the concordance (that means the percentage of patients identically classified from frozen and FFPE samples) for each combination of cut-offs between 6% and 16% for PSQ and between 13% and 23% for sqMS-PCR. With the two different optimized cut-offs for PSQ, 92% of patients were identically classified, while 8 patients out of 95 exhibited conflicting results. When using sqMS-PCR, 87% of patients were identically classified, while 12 patients out of 93 displayed conflicting results. At a cut-off of 12% or 13% for PSQ, 98% of patients had identical methylation status, irrespective of the type of sample used in the analysis. The optimized cut-off of 13% for sqMS-PCR, allowed the best concordance of 91%. Utilizing these two cut-offs on the 88 samples tested using two techniques on both types of tumor specimen, 85% of cases were concordant (Supplementary Table S1).

The optimized cut-offs, the cut-offs allowing the best concordance between frozen and FFPE samples, and the cut-off of 8% for PSQ, as previously published in our retrospective series of GBM samples [10], were all included in the analysis. As shown in Tables 2, the higher optimal AUCROC values for OS and PFS, were obtained by using PSQ on FFPE or frozen samples whatever the cut-offs tested, except using FFPE samples with the cut-off optimized on frozen samples (6% in this series) which resulted in a lower value.

**Figure 4:**
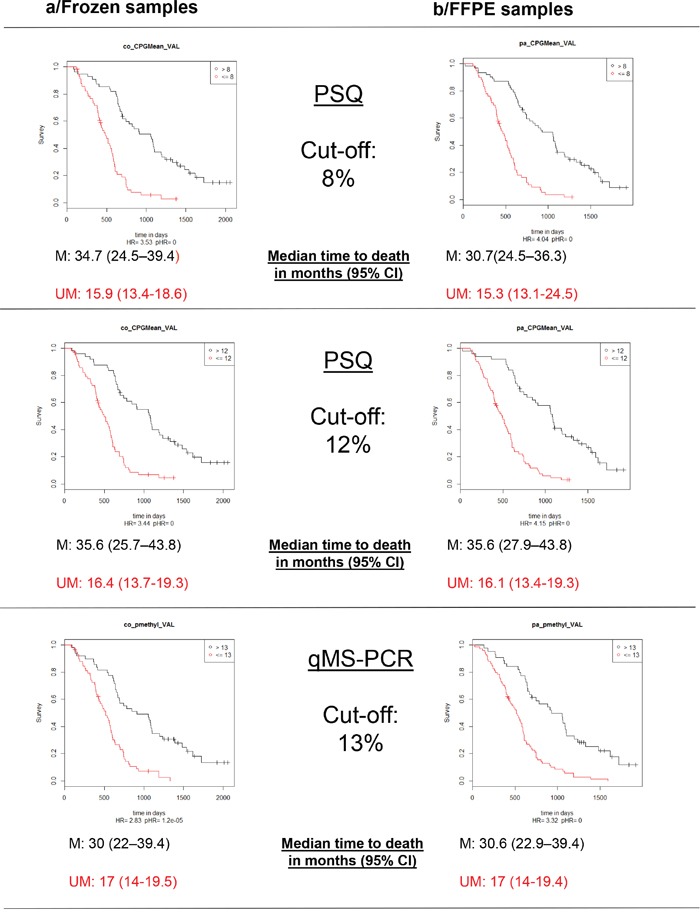
Kaplan-Meier analysis of overall survival (OS) according to MGMT promoter methylation status M: patients with a value above the calculated cut-off and therefore considered as methylated; UM: patients with a value below or equal to the calculated cut-off and therefore considered as unmethylated.

**Figure 5:**
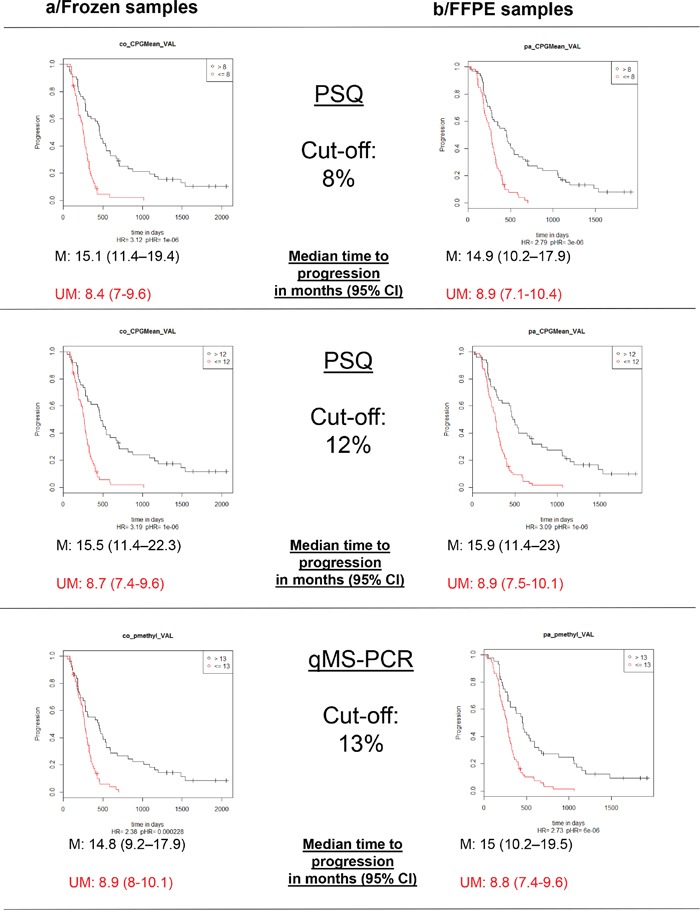
Kaplan-Meier analysis of progression free survival (PFS) according to MGMT promoter methylation status M: patients with a value above the calculated cut-off and therefore considered as methylated; UM: patients with a value below or equal to the calculated cut-off and therefore considered as unmethylated.

**Figure 6:**
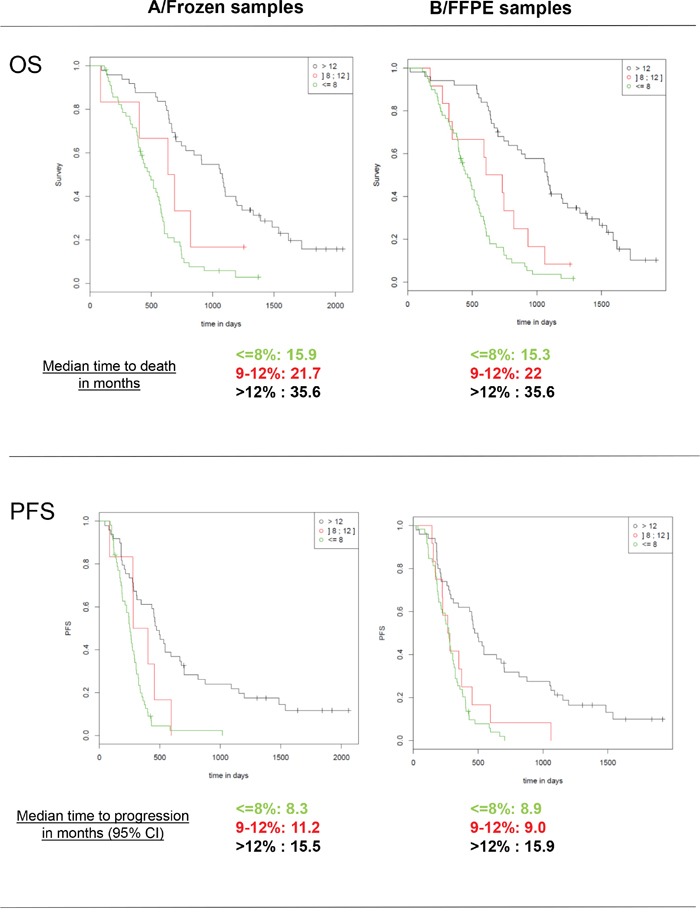
Kaplan-Meier analysis of overall survival (OS) and progression free survival (PFS) according to MGMT promoter methylation status tested by PSQ with a classification in three groups: “unmethylated” (0-8%), “methylated” (13-100%) and a grey zone for patients with intermediate values (9-12%)

The percentages of patients classified as “methylated” when employing a cut-off of 8% for PSQ were 49% for frozen samples and 51% for FFPE samples. Moreover, when utilizing a cut-off of 12%, the results were 44 and 41%, respectively. Using the sqMS-PCR method at a cut-off of 13% resulted in 45% of patients being classified as “methylated” in the frozen samples set and 37% in the FFPE sample set (Table 2). Kaplan-Meier survival curves displaying the OS and PFS of patients dichotomized according to these cut-offs are presented in Figure 4 and 5. Further evaluation of the optimal cut-off for PSQ between 8% and 12% was investigated by examining the Kaplan Meier survival plots for patients categorized in three groups: (0%-8%), (9%-12%) and (13-100%). Patients with methylation between 9-12% had a better OS than patients with a methylation of less than or equal to 8%. A similar trend was observed for PFS, however only when the analysis was performed on frozen samples (Figure 6).

## DISCUSSION

The French National Cancer Institute (INCa) and the French Ministry of Health have established a national network of regional molecular genetics centers, located throughout the country, to provide patient access to effective molecular testing. Therefore, the selected tests are required to be robust and reproducible (i.e. give comparable results in all laboratories). This prospective study was designed to validate a test method that allows for the quantification of *MGMT* methylation in routine clinical practice. PSQ and sqMS-PCR were two methods chosen to pursue after a preliminary retrospective study [10]. Both techniques evaluated in this study, resulted in good interlaboratory reproducibility for two of three methylated cell lines tested. The level of methylation of the third cell line was comparable to the limit of quantification previously established at 4% for PSQ [10] and 15% for sqMS-PCR [17], which explains the lower interlaboratory reproducibility. For this control, reproducibility was acceptable for the PSQ method. However; this was not the case for the sqMS-PCR method. These data strengthen the high interlaboratory reproducibility of PSQ with the commercial assay used for this study, even for low levels of methylation. To our knowledge, this is the first analysis of this type that has been performed in multiple laboratories. Previously, a similar conclusion for the PSQ method had been determined by Preusser et al after comparing results obtained from two independent laboratories [18].

Additionally, the parallel analysis of frozen and FFPE samples was a unique feature to the study. PSQ and sqMS-PCR were feasible in 81% (112/139) and 78% (108/139) of cases on frozen samples and in 87% (121/139) and 85% (118/139) of cases on FFPE samples. The two major reasons preventing the analysis of frozen samples were the quality criteria concerning the minimal percentage of tumor cells (for 12% (15/127) of cases) and the lack of samples (for 9% (12/139) of cases). Moreover, the two reasons preventing analysis of FFPE samples were the lack of samples and failure analysis (7 cases with PSQ/8 cases with sqMS-PCR). It is worth mentioning that the rates of failure analysis for PSQ and sqMS-PCR were not consistent among centers, indicating a possible difference of technique execution among the laboratories. At the initiation of the study, some of the centers had already implemented one technique locally to analyze *MGMT* methylation. In our personal experience on over 200 FFPE-GBM samples, failure analysis was only observed with PSQ on older samples. For these cases, PSQ resulted in non-reproducible, false-positive results (unpublished data). In these particular cases of archived specimens with inconsistent quality dependent on fixation and storage conditions, it is strongly recommended to perform a DNA quality control check prior to the analysis. Overall, we confirm that FFPE samples are appropriate for MGMT analysis of GBM patients.

Cut-offs of 12% or 13% for PSQ and 13% for sqMS-PCR allowed the best concordance values of 98% and 91% respectively to dichotomize patients as “methylated” and “unmethylated”. Recently, high concordance results using the PSQ method in comparison to the MS-PCR method, on both frozen and FFPE tissues, has been reported [19]. Globally a convincing correlation was observed between the values obtained with the two types of samples, either with PSQ or sqMS-PCR, highlighting the intratumoral homogeneity of MGMT promoter methylation, as previously described using serial stereotactic GBM samples [20].

One of the major objectives of this study was to establish optimal predictive cut-off values. To our knowledge, this is the first time that such values have been optimized on frozen and paired FFPE samples on a prospective cohort of patients. Using the sqMS-PCR method a previous study recommended to classify as «unmethylated» samples with a ratio lower than 15% (<15%) and a ratio of 30% identified GBM patients with a long PFS [17]. Remarkably, our results showed that 13% (less than or equal to 13%) allowed for the best concordance of classification between frozen and FFPE samples as well as the best distinction for survival when considering results on frozen samples; which validates a cut-off of approximately 13%-15% to dichotomize patients. However, poor interlaboratory reproducibility was observed at these values. Analytical performances must therefore be carefully examined by each laboratory before utilizing this technique in a clinical setting. Furthermore a classification of three groups can be recommended, particularly with FFPE samples: «unmethylated» samples (ratio <=13%), samples with low methylation (between 14-23%), “methylated” samples (>23%).

In a recent review about *MGMT* methylation pyrosequencing in glioblastoma, more than 20 studies have been reported. In the majority of cases, the thresholds were between 8 and 10% [21]. Our prospective study validates a cut-off of 8% to predict poor response to TMZ treatment. It is interesting to notice that there have not been any survival improvements in the past years on patients lacking a significant level of MGMT methylation. In our retrospective cohort of 100 patients having received the standard “Stupp” protocol between 2004 and 2007, “unmethylated” patients (<=8%) had a median PFS of 9 months and a median OS of 15.7 months, which is comparable to the values obtained for the “unmethylated” patients of this study treated between 2009 and 2011 (8.4 and 15.9 months respectively). Additionally, “methylated” patients had comparable PFS (14.6 and 15.1 months) but a large improvement was observed for OS, which was 26.2 months in the previous study and 34.7 months in the current one. This data indicates that “methylated” patients may significantly benefit from new second-line treatments when compared to “unmethylated” patients. Because higher thresholds performed better when analyzing FFPE samples, we proposed as a second cut-off the value of 12%, at which an excellent concordance was observed between FFPE and frozen samples. Patients above 12% could be considered as “methylated”, while patients between 9 and 12% may be considered to have a moderate/low methylation pattern. The intermediate values pose a challenge as no conclusive evidence can be interpreted in this study to conclude a clear benefit from TMZ treatment, although these patients did present a better OS than “unmethylated” patients. This interval of values could therefore be considered a “grey zone”.

In summary, we performed a prospective multicentre trial in newly-diagnosed GBM patients homogeneously treated. Our data indicated that MGMT status can be reliably evaluated on both FFPE or frozen samples in local laboratories. There are advantages to investigating FFPE samples in routine clinical practice: samples are almost always available and selection of non necrotic areas can be easily performed. Furthermore that helps to preserve fresh frozen brain samples for alternative purposes. sqMS-PCR is a viable option for MGMT testing, particularly if there is a lack of a pyrosequencer. However, our data demonstrate PSQ is the ideal choice because of its robustness, which was shown by the strong interlaboratory reproducibility. Additionally, the PSQ method offers increased sensitivity and several independent studies have been concordant in the threshold levels that discriminate between methylated and non-methylated patients.

## MATERIALS AND METHODS

### Patients

Patients were enrolled for this study between the dates of March 11, 2009 and June 29, 2011 from 8 French centers. Eligible patients had histologically confirmed de novo-glioblastoma, were between the ages of 18-70, presented with no contraindications as dictated by the Stupp protocol and were not included in experimental therapeutic protocols. Histological diagnoses were confirmed centrally by 3 pathologists: DFB, EL and DC. The protocol was approved by the Rennes medical ethics committee and informed consent was obtained from each patient.

### *MGMT* promoter methylation analysis

In a first step of the protocol, Standard Operating Procedure (SOP) for the determination of *MGMT* promoter methylation were sent to the different centers as well as 10 quality control samples: 5 samples with DNA extracted from peripheral blood mononuclear cells and from 4 primary cell lines and 5 samples with the same bisultite converted DNA. This step allowed for the standardization of the process throughout the multiple centers. Additionally, DNA extracted from 3 primary cell lines (RNS85, RNS96 and RNS175) were used as external quality controls in each of the centers and were tested in each series of tests. The coefficient of variation (CV) was calculated to determine reproducibility.

DNA extractions from clinical samples, as well as sodium bisulfite treatment, were performed in each center according to local procedures. Frozen samples with a histologically estimated tumor cell content below 40% were excluded from the study. In regards to FFPE samples, if necessary, macro-dissection was performed to enrich tumor cell content.

PSQ was performed as previously described [10, 12] using the PyroMark CpG MGMT kit (ref. 972032, Qiagen, France). All assays were performed in duplicate and each result was averaged together. The average percentage of the 5 CpGs tested was considered. sqMS-PCR was performed with primers specific for either “methylated” or “unmethylated” DNA. Forward primers were labeled at their 5′ end with a fluorescent reporter dye (FAM), as previously described [17]. The PCR products corresponding to the “methylated” sequences have a size of 82bp while the “unmethylated” sequences have 12 additional nucleotides (94bp). Both fragments were amplified in the same reaction and PCR products were analyzed by capillary electrophoresis. Estimation of the amount of methylated DNA was calculated with the following formula, abbreviations are as follows; MF-“methylated” fraction, UM-“unmethylated” fraction:

(peak height of the MF/peak height of the UM + MF) × 100.

### Statistical analysis

Statistical analysis was performed using R statistical software (version 2.13.0, http://www.Rproject.org). Optimal risk cut-offs were determined as previously described with age and performance status introduced as adjustment factors [10]. The function risksetAUC (package risksetROC) in the R statistical software was used to obtain the area under the ROC curve. Additionally, the Harrell's C index [22] was calculated using the validate function (in Design package). To study OS and PFS, cumulative event curves (censored endpoints) were established using the Kaplan-Meier method.

## SUPPLEMENTARY TABLES





## References

[1] Wick W, Weller M, van den Bent M, Sanson M, Weiler M, von Deimling A, Plass C, Hegi M, Platten M, Reifenberger G (2014). MGMT testing—the challenges for biomarker-based glioma treatment. Nat Rev Neurol.

[2] Weller M, Stupp R, Reifenberger G, Brandes AA, van den Bent MJ, Wick W, Hegi ME (2010). MGMT promoter methylation in malignant gliomas: ready for personalized medicine?. Nat Rev Neurol.

[3] Weller M, van den Bent M, Hopkins K, Tonn JC, Stupp R, Falini A, Cohen-Jonathan-Moyal E, Frappaz D, Henriksson R, Balana C, Chinot O, Ram Z, Reifenberger G (2014). EANO guideline for the diagnosis and treatment of anaplastic gliomas and glioblastoma. Lancet Oncol.

[4] Gilbert MR, Wang M, Aldape KD, Stupp R, Hegi ME, Jaeckle KA, Armstrong TS, Wefel JS, Won M, Blumenthal DT, Mahajan A, Schultz CJ, Erridge S (2013). Dose-dense temozolomide for newly diagnosed glioblastoma: a randomized phase III clinical trial. J Clin Oncol.

[5] Stupp R, Hegi ME, Gorlia T, Erridge SC, Perry J, Hong YK, Aldape KD, Lhermitte B, Pietsch T, Grujicic D, Steinbach JP, Wick W, Tarnawski R (2014). Cilengitide combined with standard treatment for patients with newly diagnosed glioblastoma with methylated MGMT promoter (CENTRIC EORTC 26071-22072 study): a multicentre, randomised, open-label, phase 3 trial. Lancet Oncol.

[6] Reifenberger G, Hentschel B, Felsberg J, Schackert G, Simon M, Schnell O, Westphal M, Wick W, Pietsch T, Loeffler M, Weller M (2012). Predictive impact of MGMT promoter methylation in glioblastoma of the elderly. Int J Cancer.

[7] Christians A, Hartmann C, Benner A, Meyer J, von Deimling A, Weller M, Wick W, Weiler M (2012). Prognostic value of three different methods of MGMT promoter methylation analysis in a prospective trial on newly diagnosed glioblastoma. PLoS One.

[8] Dunn J, Baborie A, Alam F, Joyce K, Moxham M, Sibson R, Crooks D, Husband D, Shenoy A, Brodbelt A, Wong H, Liloglou T, Haylock B (2009). Extent of MGMT promoter methylation correlates with outcome in glioblastomas given temozolomide and radiotherapy. Br J Cancer.

[9] Collins VP, Ichimura K, Di Y, Pearson D, Chan R, Thompson LC, Gabe R, Brada M, Stenning SP (2014). Prognostic and predictive markers in recurrent high grade glioma; results from the BR12 randomised trial. Acta Neuropathol Commun.

[10] Quillien V, Lavenu A, Karayan-Tapon L, Carpentier C, Labussiere M, Lesimple T, Chinot O, Wager M, Honnorat J, Saikali S, Fina F, Sanson M, Figarella-Branger D (2012). Comparative assessment of 5 methods (methylation-specific polymerase chain reaction, MethyLight, pyrosequencing, methylation-sensitive high-resolution melting, and immunohistochemistry) to analyze O6-methylguanine-DNA-methyltranferase in a series of 100 glioblastoma patients. Cancer.

[11] Quillien V, Lavenu A, Sanson M, Legrain M, Dubus P, Karayan-Tapon L, Mosser J, Ichimura K, Figarella-Branger D (2014). Outcome-based determination of optimal pyrosequencing assay for MGMT methylation detection in glioblastoma patients. J Neurooncol.

[12] Karayan-Tapon L, Quillien V, Guilhot J, Wager M, Fromont G, Saikali S, Etcheverry A, Hamlat A, Loussouarn D, Campion L, Campone M, Vallette FM, Gratas-Rabbia-Re C (2010). Prognostic value of O6-methylguanine-DNA methyltransferase status in glioblastoma patients, assessed by five different methods. J Neurooncol.

[13] Vlassenbroeck I, Califice S, Diserens AC, Migliavacca E, Straub J, Di Stefano I, Moreau F, Hamou MF, Renard I, Delorenzi M, Flamion B, DiGuiseppi J, Bierau K (2008). Validation of real-time methylation-specific PCR to determine O6-methylguanine-DNA methyltransferase gene promoter methylation in glioma. J Mol Diagn.

[14] Sasai K, Nodagashira M, Nishihara H, Aoyanagi E, Wang L, Katoh M, Murata J, Ozaki Y, Ito T, Fujimoto S, Kaneko S, Nagashima K, Tanaka S (2008). Careful exclusion of non-neoplastic brain components is required for an appropriate evaluation of O6-methylguanine-DNA methyltransferase status in glioma: relationship between immunohistochemistry and methylation analysis. Am J Surg Pathol.

[15] Viray H, Li K, Long TA, Vasalos P, Bridge JA, Jennings LJ, Halling KC, Hameed M, Rimm DL (2013). A prospective, multi-institutional diagnostic trial to determine pathologist accuracy in estimation of percentage of malignant cells. Arch Pathol Lab Med.

[16] Smits AJ, Kummer JA, de Bruin PC, Bol M, van den Tweel JG, Seldenrijk KA, Willems SM, Offerhaus GJ, de Weger RA, van Diest PJ, Vink A (2014). The estimation of tumor cell percentage for molecular testing by pathologists is not accurate. Mod Pathol.

[17] Nguyen A, Legrain M, Noel G, Coca A, Meyer Ea N, Schott R, Lasthaus C, Chenard MP, Gaub MP, Lessinger JM, Guenot D, Entz-Werle N (2015). An Innovative Fluorescent Semi-quantitative Methylation-specific PCR Method for the Determination of MGMT Promoter Methylation is Reflecting Intra-tumor Heterogeneity. Curr Cancer Drug Targets.

[18] Preusser M, Berghoff AS, Manzl C, Filipits M, Weinhausel A, Pulverer W, Dieckmann K, Widhalm G, Wohrer A, Knosp E, Marosi C, Hainfellner JA (2014). Clinical Neuropathology practice news 1-2014: pyrosequencing meets clinical and analytical performance criteria for routine testing of MGMT promoter methylation status in glioblastoma. Clin Neuropathol.

[19] Lattanzio L, Borgognone M, Mocellini C, Giordano F, Favata E, Fasano G, Vivenza D, Monteverde M, Tonissi F, Ghiglia A, Fillini C, Bernucci C, Merlano M (2015). MGMT promoter methylation and glioblastoma: a comparison of analytical methods and of tumor specimens. Int J Biol Markers.

[20] Grasbon-Frodl EM, Kreth FW, Ruiter M, Schnell O, Bise K, Felsberg J, Reifenberger G, Tonn JC, Kretzschmar HA (2007). Intratumoral homogeneity of MGMT promoter hypermethylation as demonstrated in serial stereotactic specimens from anaplastic astrocytomas and glioblastomas. Int J Cancer.

[21] Bienkowski M, Berghoff AS, Marosi C, Wohrer A, Heinzl H, Hainfellner JA, Preusser M (2015). Clinical Neuropathology practice guide 5-2015: MGMT methylation pyrosequencing in glioblastoma: unresolved issues and open questions. Clin Neuropathol.

[22] Harrell FE, Lee KL, Mark DB (1996). Multivariable prognostic models: issues in developing models, evaluating assumptions and adequacy, and measuring and reducing errors. Stat Med.

