# Supplementary material for: Validation of the high-performance of pyrosequencing for clinical MGMT testing on a cohort of glioblastoma patients from a prospective dedicated multicentric trial
Source: Oncotarget. 2016 Aug 17;7(38):61916–29. doi: 10.18632/oncotarget.11322 (PMC5308700; doi:10.18632/oncotarget.11322)
Supplement: Supplementary file 1 [file oncotarget-07-61916-s001.pdf]

Validation of the high-performance of pyrosequencing for clinical *MGMT* testing on a cohort of glioblastoma patients from a prospective dedicated multicentric trial

SUPPLEMENTARY TABLE

Supplementary Table S1: Results for the 88 samples tested with the two techniques on both types of tumor specimen

See Supplementary File 1
